# ZMIZ1 and estrogen receptor **α** form an essential partnership in endometrial biology

**DOI:** 10.1172/JCI199976

**Published:** 2025-12-01

**Authors:** Md Saidur Rahman, Kyeong A So, Jae-Wook Jeong

**Affiliations:** 1 Department of Obstetrics, Gynecology & Women’s Health, University of Missouri School of Medicine, Columbia, Missouri, USA.; 2 Department of Obstetrics and Gynecology, Konkuk University School of Medicine, Seoul, Republic of Korea.

## Abstract

Estrogen receptor α (ESR1) is a pivotal regulator of endometrial homeostasis and reproductive function, yet the coregulators that fine tune its transcriptional activity remain incompletely defined. In this issue of the *JCI*, Hewitt et al. identified Zinc finger MIZ-type containing 1 (ZMIZ1) as an ESR1 coregulator that is essential for stromal proliferation, decidualization, and overall endometrial integrity. ZMIZ1 deficiency was associated with endometriosis and endometrial cancer, and conditional ablation of *Zmiz1* using the *Pgr^Cre^* mouse led to infertility and accelerated fibrosis due to impaired estrogen responsiveness. These findings position ZMIZ1 as a key modulator of estrogen signaling with translational potential as both a biomarker and a therapeutic target in uterine disorders.

The endometrium, the inner epithelial layer of the uterus, is subject to regular proliferative phases as part of the menstrual cycle, and, in pregnancy, it undergoes a differentiation process known as decidualization that supports the formation of a placenta. Endometrial dysfunction contributes to infertility and pregnancy complications, endometriosis, and uterine cancers, among other conditions. Thus, the endometrium and endometrial homeostasis play a central role in female health, extending beyond the reproductive years. Remodeling of the endometrium is regulated, to a large degree, by the ovarian hormones estrogen and progesterone and their respective receptors, estrogen receptor α (ESR1) and progesterone receptor (PGR). In this issue of the *Journal of Clinical Investigation*, Hewitt et al. (1) identified ZMIZ1 as a critical coregulator of ESR1 in the uterus (Figure 1). The finding that ZMIZ1 resides within an ESR1 super-enhancer and colocalizes with ESR1 in both endometrial epithelial and stromal compartments places this factor at the center of estrogen signaling networks in reproductive tissues.

## ZMIZ1: a selective estrogen coregulator in the uterus

ZMIZ family proteins regulate diverse signaling pathways, including those mediated by the androgen receptor, p53, SMAD3/4, WNT/β-catenin, and NOTCH1 (2). Among them, ZMIZ1 is a transcriptional coactivator with established roles in development, immune regulation, and cancer progression. In the present work, conditional ablation of *Zmiz1* in *Pgr*-expressing cells of the uterus, pituitary gland, and ovary (*Zmiz1^d/d^* mice) markedly attenuated estrogen-induced transcriptional responses (1), including the expression of cell cycle regulators such as CCNA2 and FOXM1, which are essential for stromal proliferation and the decidual transition (3, 4). Notably, these effects were observed only following acute estrogen stimulation, underscoring ZMIZ1’s role as a ligand-dependent coregulator and selective amplifier of ESR1-driven gene programs. Unlike canonical coactivators such as SRC family members or CBP/p300, which broadly potentiate steroid receptor activity (5), ZMIZ1 fine tunes gene-specific transcriptional outputs, preferentially amplifying estrogen-responsive programs that govern cell cycle progression. Through this selective modulation, ZMIZ1 imparts both amplitude and specificity to ESR1 signaling in a context-dependent manner, thereby maintaining a balance between proliferative and differentiative responses during uterine remodeling.

Transcriptomic profiling in *Zmiz1^d/d^* mice further revealed that ZMIZ1 deficiency enhances SMAD/TGF-β signaling, shifting this pathway’s activity toward fibrotic remodeling in parallel with impaired estrogen responsiveness. The loss of ZMIZ1 not only compromised endometrial epithelial and stromal proliferation but also disrupted the reciprocal ESR1/PGR axis, thereby impairing the proliferative-to-decidual transition fundamental to implantation competence. By coordinating these pathways, ZMIZ1 emerges as a pivotal estrogen coregulator that safeguards uterine plasticity and reproductive capacity.

## Mechanistic insights and pathway crosstalk

The mechanistic consequences of ZMIZ1 deficiency extend far beyond a single pathway. ZMIZ1 functions as a ligand-dependent amplifier of ESR1 activity, sculpting estrogen-driven transcription in a gene-specific manner (1). In *Zmiz1*-deficient uteri, induction of E2F-dependent cell-cycle regulators such as CCNA2 and FOXM1, which are indispensable for stromal proliferation and decidual transformation (3, 4), was markedly impaired. These findings position ZMIZ1 at the critical interface between ESR1 and E2F signaling, where it ensures robust cell-cycle progression necessary for hormonally driven endometrial remodeling.

Equally important is the interplay between ZMIZ1 and the SMAD/TGF-β pathway. Hewitt et al.’s transcriptomic analyses revealed enhanced SMAD/TGF-β activity in the absence of ZMIZ1, consistent with prior evidence that this pathway restrains estrogen-induced epithelial proliferation while promoting fibrotic remodeling (6). Thus, the dual outcome of ZMIZ1 loss — diminished proliferative capacity coupled with heightened fibrotic signaling — reflects a broader imbalance that favors pathological TGF-β activity. Beyond the uterus, ZMIZ1 cooperates with diverse transcriptional regulators, including NOTCH and TP53, in breast and immune cells (7, 8), underscoring its versatility as a chromatin-associated coregulator. Taken together, these insights define ZMIZ1 as a molecular integrator that coordinates proliferative (ESR1/E2F/FOXM1) and inhibitory/fibrotic (SMAD/TGF-β) pathways. By modulating the amplitude rather than the presence of these signals, ZMIZ1 enables the endometrium to maintain a critical balance between growth, differentiation, and repair. Disruption of this balance may lead to implantation failure and contribute to the fibrotic degeneration characteristic of uterine aging.

## Limitations and future directions

While this study positions ZMIZ1 as a critical ESR1 coregulator, several questions remain unanswered. A limitation lies in the scope and translatability of the current models. The findings are derived primarily from ovariectomized, hormone-challenged mice with uterine-specific (*Pgr^Cre^*) *Zmiz1* deletion, raising questions about their relevance to human cyclical physiology, pregnancy, and endometrium. Future studies employing primary human tissues, patient-derived organoids, and cycle-matched endometrial assembloids will be essential to validate these findings in physiologically relevant contexts (9).

Mechanistically, although ZMIZ1 colocalizes with ESR1 and modulates gene-specific transcriptional programs, its precise chromatin partners and direct interactions in endometrial cells remain undefined. Elucidating these protein-protein and protein-DNA interactions will help clarify how ZMIZ1 selectively amplifies ESR1 signaling. Moreover, the observed enrichment of SMAD/TGF-β activity and fibrotic remodeling following *Zmiz1* loss is compelling but not yet proven to be causative (10). Targeted perturbations — such as pharmacologic ALK5 inhibition, genetic modulation of *Smad2/3*, or selective ligand blockade — will be required to determine whether TGF-β pathway activation directly drives the fibrotic phenotype.

Compartment-specific roles also merit further investigation. The relative contributions of stromal versus epithelial ZMIZ1 remain unresolved and could be delineated using lineage-specific or temporally controlled conditional knockout models. Such approaches will help clarify whether ZMIZ1 exerts distinct, context-dependent roles across endometrial compartments. From a translational perspective, the association of altered ZMIZ1 expression and mutation patterns with endometriosis and endometrial cancer suggests potential utility as a diagnostic biomarker (11, 12). However, prospective validation in cycle-controlled human cohorts, along with functional interrogation of cancer-associated variants, will be necessary to determine whether ZMIZ1 functions as an oncogenic amplifier, a tumor suppressor, or a context-dependent vulnerability. Finally, therapeutic opportunities centered on ZMIZ1 remain largely unexplored. Strategies aimed at modulating ZMIZ1 activity directly, altering its coregulatory interactions, or correcting downstream pathways such as E2F/FOXM1 or TGF-β signaling could translate these mechanistic insights into precision-targeted approaches for uterine disease. Addressing these knowledge gaps will be critical for translating ZMIZ1 biology from fundamental mechanism to clinical application.

## Clinical relevance: infertility, endometriosis, and endometrial cancer

The findings reported by Hewitt and colleagues highlighted pivotal role of ZMIZ1 in regulating uterine function (1). Its dysregulation had direct clinical implications across multiple gynecologic disorders, including infertility, endometriosis, and endometrial cancer. Hewitt et al. showed that *Zmiz1*-deficient mice were infertile due to disruption of the estrogen-progesterone signaling axis, impaired stromal proliferation, defective decidualization, and diminished PGR expression (1). These phenotypes closely resemble the clinical features of implantation failure and unexplained infertility (13). Consequently, altered ZMIZ1 expression or activity could serve as a molecular marker of endometrial receptivity, offering potential diagnostic and therapeutic value in infertility management.

In women with endometriosis, reduced ZMIZ1 expression in the eutopic endometrium parallels impaired stromal PGR activity, supporting its role in attenuated progesterone signaling and the development of progesterone resistance (14). Moreover, ZMIZ1’s established functions in immune regulation suggest additional relevance in the pathophysiology of primary dysmenorrhea, a frequent comorbidity of endometriosis, where immune dysregulation contributes to chronic pelvic pain and lesion progression. Indeed, *ZMIZ1* has been proposed as one of the candidate genes that may influence disease susceptibility and development (15, 16). These findings position ZMIZ1 within the molecular landscape of endometriosis and highlight its potential as both a biomarker and a therapeutic target.

Alterations in ZMIZ1 are detected in up to 14% of endometrial cancers (11). Although direct evidence for its ligand-dependent coregulator function with ESR1 in this disease remains limited, studies in ESR1-positive breast cancers have shown that ZMIZ1 participates in the ESR1 chromatin-bound complex to enhance the expression of cell cycle regulators such as E2F2. High ZMIZ1 expression correlates with poor patient outcomes, supporting its potential utility as both a prognostic marker and a therapeutic target in ESR1-positive malignancies (8). Whether ZMIZ1 exerts similar transcriptional regulatory roles in endometrial cancer remains an important question, particularly with respect to its influence on ESR1-driven gene programs.

## Broader implications and translational outlook

The identification of ZMIZ1 as a critical ESR1 coregulator carries broad implications for estrogen-driven biology well beyond the endometrium. Dysregulation of ESR1 cofactors, exemplified by ZMIZ1, may underline a spectrum of estrogen-dependent conditions, including endometriosis, endometrial cancer, and other hormone-driven disorders. Beyond reproductive biology, genome-wide association and clinical studies have linked ZMIZ1 variants to autoimmune disease, type 2 diabetes (T2D), and syndromic neurodevelopmental disorders, underscoring its pleiotropic physiological roles (17, 18). In cancer biology, ZMIZ1 has been implicated as a potential oncogene (19–21). Strong nuclear expression has been reported in 28% of breast, 15% of ovarian, and 10% of colon cancers, suggesting a conserved role across epithelial malignancies (19). Functionally, ZMIZ1 acts as a Notch1 cofactor in T-cell development and contributes to T-cell acute lymphoblastic leukemia (T-ALL), while, in breast tumors, it is coexpressed with ESR1 and enhances proliferation (21, 22). These diverse contexts support ZMIZ1 as a versatile chromatin-associated integrator, consistent with its location within an ESR1 super enhancer and its capacity to engage multiple transcriptional regulators.

From a translational perspective, ZMIZ1 holds promise as both a biomarker and a therapeutic target. In breast cancer, ZMIZ1 enhances ESR1-dependent expression of E2F2, correlating with poor patient outcomes (21). Therefore, monitoring ZMIZ1 expression could inform clinical decisions regarding implantation potential, hormonal responsiveness, or early malignant transformation (23). Therapeutically, modulating ZMIZ1 function may restore hormonal responsiveness in conditions such as endometriosis, whereas inhibition of ZMIZ1 could suppress estrogen-driven proliferation in malignancy, supported by Hewitt et al.’s finding that siZMIZ1 reduced proliferation in Ishikawa endometrial cancer cells (1).

Importantly, ZMIZ1 integrates hormonal (ESR1), immune (NOTCH, TP53), and metabolic (T2D-linked variants) signaling pathways (24), positioning it as a nodal regulator whose modulation could yield therapeutic benefits extending beyond reproductive disorders. By bridging pathways that govern endocrine, immune, and metabolic homeostasis, ZMIZ1 represents a promising translational target for systemic conditions, particularly those that disproportionately affect women.

## Conclusions

Hewitt et al. (1) establish ZMIZ1 as a pivotal coregulator of ESR1 in uterine biology, integrating estrogen-driven proliferative signals with progesterone-mediated differentiation programs. Their findings extend the current paradigm of steroid hormone action by demonstrating how loss of ZMIZ1 disrupts these intersecting pathways, resulting in impaired decidualization, infertility, fibrotic remodeling, and phenotypes reminiscent of endometriosis. Beyond identifying a previously unrecognized molecular determinant of uterine function, this work opens new avenues for research and therapeutic innovation. Future efforts should focus on defining the chromatin-level interactions and molecular partners of ZMIZ1, mapping its compartment-specific roles in stromal and epithelial populations, and validating its clinical significance in cycle-matched human tissues and disease contexts. Such advances have the potential to establish ZMIZ1 not only as a biomarker of uterine health and disease but also as a promising therapeutic entry point for the treatment of reproductive disorders and hormone-driven pathologies.

## Funding support

This work is the result of NIH funding, in whole or in part, and is subject to the NIH Public Access Policy. Through acceptance of this federal funding, the NIH has been given a right to make the work publicly available in PubMed Central.

- The Eunice Kennedy Shriver National Institute of Child Health and Human Development to JWJ.
- NIH awards P01HD106485, R01HD108895, R01HD102170, and R01HD101243 to JWJ.

## Figures and Tables

**Figure 1:**
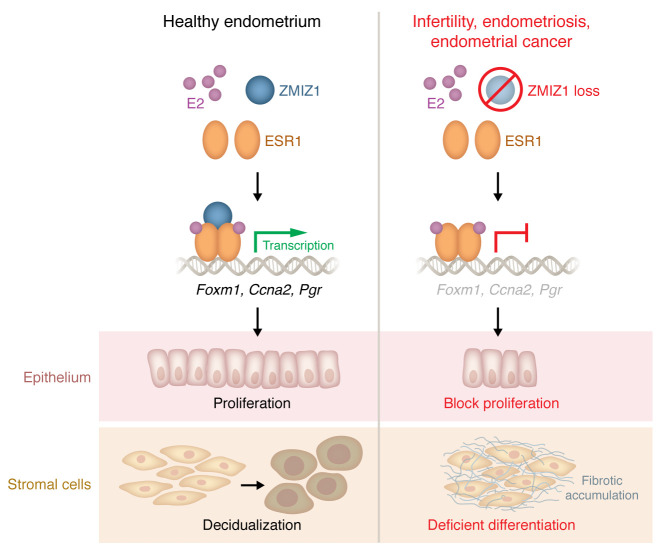
ZMIZ1 functions as a transcriptional coregulator of ESR1 to maintain estrogen-progesterone balance and uterine homeostasis. The study by Hewitt et al. reported that Zinc Finger MIZ-Type Containing 1 (ZMIZ1) cooperates with estrogen receptor-α (ESR1) to regulate estrogen-responsive transcription essential for uterine proliferation and differentiation (1). In healthy endometrium (left column): Upon estrogen stimulation, ESR1 recruits ZMIZ1 to estrogen-responsive super enhancers. The ESR1-ZMIZ1 complex amplifies transcription of key downstream genes — including Foxm1, Ccna2, and Pgr — that govern epithelial cell-cycle progression, induction of stromal progesterone receptor, and progesterone-dependent decidualization. Coordinated activation of these pathways sustains epithelial proliferation, stromal remodeling, endometrial receptivity, and fertility, preserving uterine homeostasis. In the setting of ZMIZ1 deficiency (right column): Loss or reduction of ZMIZ1 disrupts ESR1-mediated transcriptional programs, resulting in decreased Foxm1, Ccna2, and Pgr expression, defective epithelial proliferation, and impaired stromal differentiation. Dysregulated estrogen signaling enhances SMAD/TGF-β pathway activity, leading to excessive extracellular matrix deposition, collagen accumulation, and progressive age-dependent uterine fibrosis. These molecular defects culminate in infertility, attenuated decidual response, and fibrotic remodeling observed in Zmiz1d/d mice. ZMIZ1 mutations have been detected in endometrial carcinoma, and diminished ZMIZ1 expression in the eutopic endometrium of women with endometriosis is associated with infertility, endometriosis, and endometrial cancer, underscoring the translational relevance of ZMIZ1 dysregulation.

## Version 1 12/01/2025

Electronic publication
